# Prevalence and pattern of mental disability using Indian disability evaluation assessment scale in a rural community of Karnataka

**DOI:** 10.4103/0019-5545.39754

**Published:** 2008

**Authors:** S. Ganesh Kumar, Acharaya Das, P. V. Bhandary, Shashi Joyce Soans, H. N. Harsha Kumar, M. S. Kotian

**Affiliations:** Department of Community Medicine, Kasturba Medical College, Mangalore, Karnataka, India; 1 Department of Community Medicine, K. S. Hegde Medical College, Mangalore, Karnataka, India; 2 A. V. Baliga Charitable Hospital and Psychiatrist, District Govt. Hospital, Udupi district, Karnataka, India

**Keywords:** Mental disability, prevalence and pattern, rural community

## Abstract

**Background:**

In the present era, mental disability is a major public health problem in the society. Many of the mental disabilities are correctable if detected early.

**Objectives:**

To assess the prevalence and pattern of mental disability.

**Setting and Design:**

Community-based cross-sectional study. One thousand subjects in all age groups were randomly selected from four villages in Udupi district, Karnataka.

**Materials and Methods:**

The study was conducted by making house-to-house visits, interviewing, and examining all the individuals in the families selected with pre-designed and pretested questionnaire.

**Statistical Analysis:**

Proportions, chi-square test.

**Results and Conclusion:**

The prevalence of mental disability was found to be 2.3%. The prevalence was higher among females (3.1%) than among males (1.5%). The prevalence was higher among the elderly age group and illiterates. There is ample scope for community-based rehabilitation of the mentally disabled.

## INTRODUCTION

World Health Organization estimates that 10% of the world's population has some form of mental disability and 1% suffersfrom severe incapacitating mental disorders.[1] Community-based surveys conducted during the past two decades in India showed that the total prevalence of psychiatric disorder was around 5.8%.[2] In contrast, recent National Sample Survey Organization report revealed prevalence as little as 0.2%.[3]

Mental Health Act 1987 provides safeguards against stigmatization and discrimination for patients suffering from mental illness. Medical colleges will play a pivotal role in making the programs operational and monitoring them in the districts in which they are located.[4] The latest phase in the development of mental health services in India has been the community care approach. With modern health care, it is possible to treat and cure 60% of the cases with mental morbidity and to avoid disability in 80%.[5] Very few community-based studies have been conducted in India to understand the problem. Such studies will be a useful tool for developing community-based rehabilitation programs for the mentally disabled. In the above context, the present study was conducted in a rural area in Karnataka.

## MATERIALS AND METHODS

This was a community-based cross-sectional study carried out over a period of 1 year from January to December 2004. The study was conducted at the rural field-practice area of a teaching institution, which covers a population of 45,000 spread over 11 villages of a *taluka* in the state of Karnataka in India. A favorable sex ratio of 1,140 females per 1,000 males and high literacy rate of 94.1% were the striking features of this area. Four villages, namely, Kotemattu, Yenegudda, Kidiyoor, and Kadekar were covered. The population covered by these four villages was 16,298. The sample size was estimated for infinite population by using the formula 4pq/d^2^, where prevalence was taken as 10%.[1] Required precision of the estimate (d) was set at 20%. Using the above formula, the sample size was estimated to be 900. After adding nonresponse error of 10%, an additional 100 subjects were included. Thus, 1000 subjects in all age groups were selected for this study.

### Sampling

Probability proportional to size was used to select the study sample from each village. These villages have family folders containing the family particulars of each household. In the next step, in each of the four centers, all family folders were arranged in a serial order. Then, the first folder was selected randomly from the random number table, and the names of the eligible candidates from that household were noted down. Similarly, the next folder was randomly picked up, and the names of all the eligible candidates of that household were listed. This procedure was repeated till the desired number of eligible persons was achieved from each center. In this way, a complete list of all the designated households and the candidates to be interviewed was prepared before making the field visits.

The study was conducted by making house-to-house visits, interviewing, and examining all the individuals in the families selected using a pretested questionnaire. A prerequisite for the eligibility was membership in the household, defined as all persons who are biologically related with each other and eating from a common kitchen. If a designated person could not be contacted or was not cooperative during the three separate visits, the subject was considered as nonrespondent. Mental disability was assessed by Indian Disability Evaluation and Assessment Scale (IDEAS), a scale for measuring and quantifying disability in mental disorders, developed by the Rehabilitation Committee of Indian Psychiatric Society.[6] IDEAS was field-tested in nine centers all over India and has now been gazetted by the Ministry of Human Resources and Empowerment, Government of India, as the recommended instrument to measure psychiatric disability.[7] Disability in children below the age of 5 years was assessed based on the instrument designed on the lines of questionnaire taken from Action Aid India. Action Aid India instrument is used for the assessment of mental disability of a child. Children were examined and developmental delay in responding to the name or voice, smile; locomotor, communication, and learning difficulties were noted down.[8]

The data collected was tabulated and analyzed by using the Statistical Package for Social Sciences (SPSS) version 11.5 for Windows. Findings were described in terms of percentages. Chi-square test was carried out to test the differences between proportions. A probability level of less than 0.05 was considered significant.

## RESULTS

Of the 1,000 subjects enrolled into the study, 954 subjects were available for the final analysis (response rate, 95%). The overall prevalence of mental disability was found to be 2.3% (22). Among those with mental disability, majority (10) had mild disability. This was followed by severe (6), moderate (4), and profound (2) mental disability. All the disabled subjects were previously diagnosed with one or the other mental ailment, viz., affective disorders (7), mental retardation (3), fits (3), neurosis (9), schizophrenia (1), alcohol addiction (4).

The present study showed that 32% of the disabled were males, 68% were females; and the association was not significant (X^2^ = 2.81; *P* > 0.05). The prevalence of disability among the elderly group (>60 years) was high (4%).The prevalence of disability was higher among the group of persons with low socioeconomic status (3%), and the association was not found to be significant (X^2^ = 2.41; *P* > 0.05). Around one-third (32%) of the disabled were illiterates, and those with education level above 10^th^ standard had very low prevalence. As literacy level increased, the prevalence declined significantly (X^2^ = 19.52; *P* = < 0.001) [Table 1].

**Table 1:** Prevalence of disability according to socio-demographic variables

Socio-demographic variables	Subjects interviewed	Number of mental disabled	Prevalence (%)
Gender			
Male	472	7	1.5
Female	482	15	3.1
Age group (years)			
<5	72	1	1.4
5-14	122	1	0.8
15-59	635	15	2.4
60 and above	125	5	4.0
socioeconomic status			
Low	456	14	3.0
Middle	486	8	1.6
High	12	0	0.0
Literacy* (schooling)			
Illiterate	84	7	8.3
1-4	118	6	5.0
5-10	522	7	1.3
>10	125	1	0.8

*n* = 954:

* A total of 105 (10.6%) subjects are below the age of 7 years. The total number of disabled in the age group of 7 years and above was 21 (X^2^ = 19.52; *P* = < 0.001)

## DISCUSSION

Well-documented studies to determine the prevalence and pattern of mental disability are few. There are no community-based studies using IDEAS for assessment of mental disability, but there are some hospital-based studies among mental illness patients to assess mental disability using IDEAS. This instrument was used in other studies to assess mental disability in mental illness that included schizophrenia, bipolar affective disorder, anxiety disorders, depression, obsessive compulsive disorder, dementia, mental and behavioral disorders due to the intake of alcohol.[9,10] Also, the data collected by health workers could not detect mild degrees of disability because of their limited knowledge and lack of training. The widely differing prevalence rates found in various studies are due to wide difference in the samples, methods of assessment, and definitions used.

The present study showed a higher prevalence of mental disability in comparison to prevalence in general.[3,10] This is because of detection of even mild degrees of disability in our study. The prevalence was more common among the geriatric age group than among the productive age group. This has been attributed to higher prevalence of depression among the geriatric age group. Higher prevalence of mental disability among females is due to the fact that most of them were suffering from neuroses and depression. The prevalence of mental disability was lowest among the group of persons with high socioeconomic status. Disabled in this area are better educated when compared to the disabled people of other parts of country.[3]

About a third of the 7 million to 8 million Indians who suffer from psychotic disorders will be severely disabled and will require intense rehabilitation inputs.[11] Those who are moderately disabled also require intervention, largely in relation to work and employment. It is therefore critical to establish community-based centers which offer not just medical treatment but psycho-social rehabilitation as well. These centers will help in the rehabilitation of the chronic mentally disabled, creation of awareness on mental health, diagnosis of the undiagnosed mild and moderate cases, and treatment. Disability measurement is therefore important to assess the burden and plan services and welfare benefits for this group of disabled.

An apparent limitation of our study was that the possibility of generalization to include varied groups of the population is very low. Considering that the population in this study had a very high literacy rate and favorable sex ratio, it is likely that the results can be generalized to apply in similar settings. We could not interview nonrespondents because of their noncooperation or non-availability during our field visits. Since the proportion of nonrespondents was very small in our study population, we expect only a minimal effect on our prevalence estimate.
